# Effects of fetal presentation on mode of delivery in 26 143 twin pregnancies: A nationwide, population‐based observational study of 31‐year real‐world data

**DOI:** 10.1002/ijgo.70103

**Published:** 2025-03-29

**Authors:** Meryam Sugulle, Katariina Laine, Tiril Tingleff, Gulim Murzakanova, Sari Räisänen

**Affiliations:** ^1^ Division of Obstetrics and Gynaecology, Department of Obstetrics Oslo University Hospital Oslo Norway; ^2^ Faculty of Medicine University of Oslo Oslo Norway; ^3^ Norwegian Research Centre for Women's Health Oslo University Hospital Oslo Norway; ^4^ Division of Obstetrics and Gynaecology, Department of Obstetrics Haukeland University Hospital Bergen Norway; ^5^ Laurea University of Applied Sciences Vantaa Finland

**Keywords:** cesarean section, combined delivery, fetal presentation, intrapartum, twin pregnancy, vaginal delivery

## Abstract

**Objective:**

To describe the rate of combinations of fetal lies and presentations in twin pregnancies at delivery and to assess the effects of fetal lies and presentations on mode of delivery as well as fetal and maternal birth outcomes.

**Methods:**

This population‐based cohort study used real‐world data from the Medical Birth Registry of Norway and included all twin births (*n* = 26 143) in Norway during 1988–2018. Logistic Regression Analysis was Applied. Main outcome measures were planned and actual modes of delivery categorized into vaginal deliveries and cesarean sections. Secondary outcomes were postpartum hemorrhage and intrapartum stillbirth.

**Results:**

Most twin births were planned as vaginal deliveries (83.4%, 21 811/26 143), of which (65.9%, 14 371/21 811) were successful. Vaginal delivery was more often successful in parous than in nulliparous women, or if the first or both twins were in cephalic presentation compared with non‐cephalic presentation of the first or both twins. Planned cesarean section was more likely to be chosen if the first or both twins were in non‐cephalic presentation than if they were in cephalic presentation. Maternal parity, demographic and obstetric risk factors had little or no effect on the association between twin presentations and planned or actual delivery mode.

**Conclusions:**

More than half of women with a twin pregnancy had a successful vaginal delivery. Keeping the option of the vaginal route as the primary mode of delivery in a twin pregnancy maintains the right of the woman to have a vaginal delivery and avoid a uterine scar.

## 1 INTRODUCTION

Twin delivery is perceived as a challenge in modern obstetrics, and high cesarean section (CS) rates of 40%–99% are reported for many European countries and the United States.^1^, ^2^ However, there is no universal international consensus on the optimal mode of delivery for twins.

As there is a trend in many countries towards delivery by CS in the majority of singleton breech deliveries, a first or second twin in breech presentation may also be perceived as an indication for planned CS. The Norwegian National Obstetric Guidelines recommend vaginal delivery in singleton breech presentation from gestational age (GA) 34 + 0 weeks, and individual counseling before GA 34 + 0 weeks. These recommendations are also valid for twin pregnancies with the first twin in breech presentation.^3^ Obstetric practice in Norway is characterized by low overall CS rates of <17%, as well as stable CS rates in singleton breech pregnancies and in multifetal pregnancies over the past two decades.^4^, ^5^ At the same time, fetal and neonatal mortality rates are low.^4^

Previous research into twin pregnancies has mostly focused on management during pregnancy, and few previous studies have assessed the role of the twins' presentations on planned and actual delivery modes.^6^, ^7^, ^8^, ^9^ Most of these studies have been single‐center studies and/or conducted as randomized controlled trial settings, and excluded lower GAs. The aim of the present study was to describe the rate of combinations of twin fetal presentations at delivery and to identify their associations with the mode of delivery as well as fetal and maternal birth outcomes.

## 2 MATERIALS AND METHODS

Data were obtained from the Medical Birth Registry of Norway (MBRN) in accordance with the Norwegian Health Research Act. The MBRN, as a mandatory national health registry, does not require informed consent for the registration or use of anonymized data for research purposes.

All twin births at GA 22–44 weeks (*n* = 27 936) during 1988–2018 were included. Cases with information lacking on GA or fetal presentations (*n* = 793, 6.4%) were excluded, and thus the final study population included 26 143 twin births, (52 286 newborns).

Maternity services are free of charge for all women in Norway. All twin births take place in hospital, led by an obstetrician.

Both the standardized pregnancy card with information on woman's health and events during labor and delivery are registered in the hospital patient records, and the MBRN is notified directly after birth.

More than 97% of pregnant women attend the ultrasound examination at GA 17–20 weeks offered as part of the antenatal care,^5^ The ultrasonography‐based estimated date of delivery (EDD) has been registered in the MBRN since 1999. Before 1999, the EDD is based on the reported last menstrual period and calculated using Naegele's rule.

The main exposure was fetal presentation in twin pregnancies. Fetal lie was categorized into longitudinal, transverse, and oblique. Presentation was defined as the leading part of the fetus at birth, and categorized into cephalic or non‐cephalic. The combination of the two fetal presentations at delivery was classified into four categories: cephalic/cephalic, cephalic/non‐cephalic, non‐cephalic/cephalic, and non‐cephalic/non‐cephalic. In the MBRN, cephalic presentation was a dichotomized variable, and cephalic presentations other than occiput anterior are registered as “abnormal cephalic.”

All breech presentations and transverse lies were placed in the non‐cephalic group. All first twins in the transverse lie were delivered by planned CS. The main outcome variable was the mode of delivery, categorized into planned CS and planned vaginal delivery. Planned vaginal deliveries were reported as vaginal deliveries or intrapartum CS based on the actual mode of delivery, with actual vaginal deliveries interpreted as “successful.” Combined delivery was defined as vaginal delivery of the first twin, followed by intrapartum CS for the second twin. Secondary outcomes were postpartum hemorrhage (PPH) and intrapartum stillbirth.

### 2.1 Covariates

Factors associated with CS were selected based on previous reports in the literature and accounted for in analysis adjustments: GA, maternal age,^10^ maternal country of birth,^11^ pre‐eclampsia (PE), diabetes,^12^ and previous CS.^13^, ^14^

Gestational age groups were categorized, based on clinical relevance as follows: GA <34 weeks, GA 34 + 0 to 36 + 6 weeks, and GA ≥37 + 0 weeks. Maternal age in completed years at the time of delivery was categorized into five groups: <25 years, 25–29 years (reference group), 30–34 years, 35–39 years, and ≥40 years. Maternal country of birth was dichotomized into Norway (reference group) or not Norway.

Women with PE, eclampsia, or HELLP (hemolysis, elevated lever enzymes, and low platelets) syndrome were merged into a dichotomous variable (yes or no). The following MBRN definitions were used: PE (blood pressure >140/90 mm Hg and proteinuria), HELLP syndrome, and/or eclampsia (generalized seizures occurring antepartum, intrapartum, or within 7 days postpartum in combination with PE or hypertension, without a neurologic etiology).

Women with diabetes mellitus were categorized into type 1, type 2, and gestational diabetes mellitus. Gestational diabetes was defined according to national guidelines at the time of pregnancy.^15^, ^16^, ^17^, ^18^ Parity was grouped into nulliparous and parous (at least one previous delivery).

Postpartum hemorrhage was defined as more than 500 mL blood loss after birth (available from 1999).

The overall rate of missing data for all other variables (except GA or fetal presentation as specified above) was <1%.

### 2.2 Statistical analyses

Descriptive statistics were used to quantify the prevalence rates of planned CS, planned vaginal delivery, and intrapartum CS according to maternal characteristics and GA. Crude odds ratios (ORs) and adjusted ORs (aORs) with 95% confidence intervals (CI) for the delivery mode were determined in crude and multivariable logistic regression analyses.

Three models were created: model 1 was adjusted for GA only; model 2 was adjusted for GA, maternal age, and maternal country of birth; and model 3 was adjusted for GA, diabetes, and PE. Data on parous women with previous CS were included in model 3. We calculated the percentage reduction in ORs between the models using the following formula if applicable: (OR model 1 − OR model *x*)/(OR model 1 − 1).^19^ Model *x* was either model 2 or 3, and values >0 were considered to indicate a valid and significant contribution.

The 31‐year study period was categorized into six time periods (1988–1993, 1994–1998, 1999–2003, 2004–2008, 2009–2013, 2014–2018) to calculate rates in planned and actual delivery modes.

Frequencies of intrapartum stillbirth by delivery mode, GA, twin birth order, and presentation were reported in numbers. Statistical analyses were performed with IBM SPSS Statistics for Windows (version 26, IBM Corporation, Armonk, NY, USA). *P*‐values less than 0.05 were considered statistically significant.

### 2.3 Ethical approval

This study is part of the PURPLE Study approved by the Regional Committee for Medical Research Ethics in South East Norway (Ref 2015/681; March 24, 2015, latest renewal: September 14, 2023) and the Institutional Personal Data Officer at Oslo University Hospital.

## 3 RESULTS

The study population comprised 26 143 twin births. Table 1 lists the rates of fetal lies and presentation groups at birth. When combining cephalic and abnormal cephalic presentations into one group, most first twins were in cephalic presentation (20 514, 78.5%), and more than half of both twins were in cephalic presentation (13 626, 52.1%). In approximately 1 in 10 pregnancies, both twins were in non‐cephalic presentation (3348, 12.8%).

**TABLE 1:** Rates of twin fetal lies and presentation (*n* = 26 143 pregnancies). Births before gestational week 22 and after gestational age 43 weeks were excluded. Cases with missing information on presentation were removed.

First twin	Second twin			
	Cephalic	Breech	Abnormal cephalic^a^	Transverse
	*n* (%)	*n* (%)	*n* (%)	*n* (%)
Cephalic	12 026 (46.0)	6038 (23.1)	1009 (3.9)	574 (2.2)
Breech	2105 (8.1)	2554 (9.8)	118 (0.5)	522 (2.0)
Abnormal cephalic^a^	392 (1.5)	243 (0.9)	199 (0.8)	33 (0.1)
Transverse	53 (0.2)	50 (0.2)	5 (0.02)	222 (1.0)

^a^ Abnormal cephalic: term used in the Medical Birth Registry of Norway registration form, implying all cephalic presentations other than occiput anterior.

### 3.1 Planned mode of delivery

Most twin pregnancies were planned as vaginal deliveries (*n* = 21 811, 83.4%), with the remaining one in six planned as CSs (*n* = 4332, 16.6%). The proportions of nulliparous and parous women with planned vaginal twin birth were almost equal (*n* = 9321 [82.4%] vs. *n* = 12 490 [84.2%], respectively) (Table 2). Non‐cephalic presentation of the first or both twins was more likely to result in planned CS (*n* = 770 [33.8%] and *n* = 1365 [40.8%], respectively) compared with first or both twins in cephalic presentation (*n* = 743 [10.8%] and *n* = 1454 [10.7%], respectively) (Table 2). The associations between mode of planned delivery, maternal and delivery characteristics, and pregnancy complications are presented in Table 2.

**TABLE 2:** Planned and actual mode of birth according to twin presentations, and maternal and obstetric factors.

Entire study population (*N* = 26 143)	Planned CS (16.6%, *n* = 4332)	Planned vaginal delivery (83.4%, *n* = 21 811)	Vaginal delivery, both twins (65.9%, 14 371/21 811)	Intrapartum cesarean, both twins (30.9%, 6729/21 811)	Combined delivery, first twin vaginal, second twin CS (3.3%, 711/21 811)
Combination of presentations					
Nulliparous (*n* = 11 309)	17.6 (1988)	82.4 (9321)	56.5 (5269)	40.7 (3795)	2.8 (257)
Cephalic/cephalic (*n* = 5987)	10.7 (640)	89.3 (5347)	64.2 (3433)	33.4 (1788)	2.4 (126)
Cephalic/non‐cephalic (*n* = 2793)	11.1 (309)	88.9 (2484)	62.6 (1554)	32.5 (808)	4.9 (122)
Non‐cephalic/cephalic (*n* = 1046)	38.5 (403)	61.5 (643)	23.3 (150)	76.5 (492)	<5 cases
Non‐cephalic/non‐cephalic (*n* = 1483)	42.9 (636)	57.1 (847)	15.6 (132)	83.5 (707)	0.9 (8)
Parous (*n* = 14 834)	15.8 (2344)	84.2 (12 490)	72.9 (9102)	23.5 (2934)	3.6 (454)
Cephalic/cephalic (*n* = 7639)	10.7 (814)	89.3 (6825)	80.1 (5470)	17.1 (1165)	2.8 (190)
Cephalic/non‐cephalic (*n* = 4095)	10.6 (434)	89.4 (3661)	77.6 (2842)	16.1 (590)	6.3 (229)
Non‐cephalic/cephalic (*n* = 1235)	29.7 (367)	70.3 (868)	50.5 (438)	47.8 (415)	1.7 (15)
Non‐cephalic/non‐cephalic (*n* = 1865)	39.1 (729)	60.9 (1136)	31.0 (352)	67.3 (764)	1.8 (20)
GA (wk)					
Nulliparous					
<34 (*n* = 2414)	9.7 (235)	90.3 (2179)	45.9 (1001)	51.9 (1131)	2.2 (47)
34–36 (*n* = 3627)	17.2 (623)	82.8 (3004)	57.6 (1730)	39.7 (1192)	2.7 (82)
≥37 (*n* = 5268)	21.5 (1130)	78.5 (4138)	61.3 (2538)	35.6 (1472)	3.1 (128)
Parous					
<34 (*n* = 1767)	12.1 (213)	87.9 (1554)	45.3 (704)	51.2 (795)	3.5 (55)
34–36 (*n* = 4349)	13.4 (581)	86.6 (3768)	69.1 (2603)	27.4 (1033)	3.5 (132)
≥37 (*n* = 8718)	17.8 (1550)	82.2 (7168)	80.8 (5795)	15.4 (1106)	3.7 (267)
Maternal age in years					
<25 (*n* = 2838)	12.1 (343)	87.9 (2495)	66.1 (1648)	30.9 (771)	3.0 (76)
25–29 (*n* = 7573)	14.7 (1115)	85.3 (6458)	67.3 (4346)	30.0 (1936)	2.7 (176)
30–34 (*n* = 9612)	16.8 (1611)	83.2 (8001)	66.8 (5348)	29.6 (2369)	3.5 (284)
35–39 (*n* = 5121)	19.6 (1002)	80.4 (4119)	64.3 (2647)	32.0 (1317)	3.8 (155)
≥40 (*n* = 999)	26.1 (261)	73.9 (738)	51.8 (382)	45.5 (336)	2.7 (20)
Maternal country of birth					
Norway (*n* = 21 040)	16.1 (3388)	83.9 (17 652)	66.6 (11 754)	30.1 (5311)	3.3 (587)
Not‐Norwegian (*n* = 5103)	18.5 (944)	81.5 (4159)	62.9 (2617)	34.1 (1418)	3.0 (124)
PE/Eclampsia/HELLP (*n* = 2759)	19.0 (524)	81.0 (2235)	49.6 (1109)	47.3 (1057)	3.1 (69)
Diabetes type 1 (*n* = 97)	24.7 (24)	75.3 (73)	49.3 (36)	50.7 (37)	0
Diabetes type 2 (*n* = 131)	22.9 (30)	77.1 (101)	50.5 (51)	46.5 (47)	<5 cases
Gestational diabetes (*n* = 574)	19.7 (113)	80.3 (461)	59.4 (274)	36.4 (168)	4.1 (19)
Parity					
Nulliparous (*n* = 11 309)	17.6 (1988)	82.4 (9321)	56.5 (5269)	40.7 (3795)	2.8 (257)
Parous (*n* = 14 834)	15.9 (2344)	84.2 (12 490)	72.9 (9105)	23.5 (2934)	3.6 (454)
Parous women with previous CS^a^ (*n* = 2155)	38.2 (824)	61.8 (1331)	40.3 (536)	56.9 (758)	2.8 (37)

Abbreviations: CS, cesarean section; GA, gestational age; HELLP, hemolysis elevated liver enzymes low platelets syndrome; PE, pre‐eclampsia.

^a^ Calculated of 14 834 parous women only.

Compared with cephalic presentation of both twins, aORs for planned CS for nulliparous women were 6.74 (95% CI 5.89–7.70) when both twins were in non‐cephalic presentation, and 5.20 (95% CI 4.47–6.05) when only the first twin was in non‐cephalic presentation. For parous women, aORs for planned CS were 5.55 (95% CI 4.93–6.25) when both twins were in non‐cephalic presentation and 3.56 (95% CI 3.09–4.11) when only the first twin was in non‐cephalic presentation (Table 3, model 1).

**TABLE 3:** Crude and adjusted odds ratio (OR, aOR) for planned cesarean section in the entire study population (*N* = 26 143).

	Crude OR	Model 1 aOR	Model 2 aOR	Difference with model 1^a^ (%)	Model 3 aOR	Difference with model 1 (%)^a^
	(95% CI)	(95% CI)	(95% CI)		(95% CI)	
	*P*‐value	*P*‐value	*P*‐value		*P*‐value	
Combination of presentations						
Nulliparous (*n* = 11 309)						
Cephalic/cephalic	Ref	Ref	Ref		Ref	
Cephalic/non‐cephalic	1.04 (0.90–1.20) 0.600	1.09 (0.94–1.26) 0.262	1.06 (0.92–1.23) 0.431	^b^	1.09 (0.94–1.26) 0.263	^b^
Non‐cephalic/cephalic	5.24 (4.51–6.08) <0.001	5.20 (4.47–6.05) <0.001	5.23 (5.83–7.64) <0.001	<0	5.21 (4.48–6.06) <0.001	<0
Non‐cephalic/non‐cephalic	6.27 (5.50–7.16) <0.001	6.74 (5.89–7.70) <0.001	6.68 (5.83–7.64) <0.001	1.04	6.76 (5.91–7.74) <0.001	<0
Parous (*n* = 14 834)						
Cephalic/cephalic	Ref	Ref	Ref		Ref	
Cephalic/non‐cephalic	0.99 (0.88–1.12) 0.923	1.00 (0.88–1.13) 0.993	0.98 (0.87–1.11) 0.739	^b^	1.04 (0.91–1.18) 0.585	^b^
Non‐cephalic/cephalic	3.55 (3.08–4.09) <0.001	3.56 (3.09–4.11) <0.001	3.61 (3.13–4.17) <0.001	<0	3.83 (3.30–4.46) <0.001	<0
Non‐cephalic/non‐cephalic	5.38 (4.78–6.06) <0.001	5.55 (4.93–6.25) <0.001	5.57 (4.94–6.27) <0.001	<0	5.98 (5.27–6.78) <0.001	<0

*Note*: Model 1: adjusted for gestational age; model 2: adjusted for gestational age, maternal age, and maternal country of birth; model 3: adjusted for gestational age, diabetes, and pre‐eclampsia. For parous women, previous cesarean included in model 3.

Abbreviation: CI, confidence interval; Ref., reference group.

^a^ The percentage differences between models 1 and 2, and models 1 and 3 were calculated by the formula (OR model 1 − OR model *x*)/(OR model 1 − 1).

^b^ As the aORs were not significantly different between models 1, 2, and 3 for cephalic/non‐cephalic presentation, no calculation on difference was appropriate.

The findings for the partially adjusted logistic regression models in Table 3 indicate that GA, maternal age, maternal country of birth, diabetes, PE, and previous CS had no or a minor effect on the association between twin presentation and planned CS. It appeared that in nulliparous women, maternal age and country of birth explained 1.04% of the association between non‐cephalic presentation of both twins and planned CS birth. Consequently, it seems that fetal presentation was the most important factor in the decision for planned CS (models 2 and 3, Table 3).

### 3.2 Actual mode of delivery

Among the planned vaginal deliveries, 14 371 (65.9%) were successful for both twins while 6729 (30.9%) resulted in intrapartum CS for both twins. In 711 (3.3%) planned vaginal deliveries, the first twin was born vaginally and the second twin by CS—that is, combined delivery (Table 2).

Planned vaginal deliveries were successful for both twins in 5269 (56.5%) of the nulliparous women and 9102 (72.9%) of the parous women. Vaginal delivery occurred in 536 (40.3%) of the 2155 women with previous CS and a planned vaginal delivery (Table 2).

Vaginal deliveries were successful in cephalic presentation of the first or both twins in 4987 (63.7%) of the nulliparous women and 8312 (79.3%) of the parous women. The corresponding rates for nulliparous and parous women with non‐cephalic presentation of the first or both twins were 282 (18.9%) and 790 (39.4%), respectively (Table 2).

Compared with both twins in cephalic presentation, aORs for intrapartum CS for nulliparous women were 9.08 (95% CI 7.48–11.04) when both twins were in non‐cephalic presentation and 5.91 (95% CI 4.88–7.16) when only the first twin was in non‐cephalic presentation (model 1, Table 4). For parous women, aORs for intrapartum CS were 8.53 (95% CI 7.39–9.85) when both twins were in non‐cephalic presentation and 4.02 (95% CI 3.45–4.67) when only the first twin was in non‐cephalic presentation (model 1, Table 4).

**TABLE 4:** Crude and adjusted odds ratio (OR, aOR) for intrapartum cesarean section (CS) among individuals planned for vaginal delivery (*n* = 21 811).

Combination of presentations	Crude OR	Model 1 aOR	Model 2 aOR	Difference with model 1^a^ (%)	Model 3 aOR	Difference with model 1^a^ (%)
	(95% CI)	(95% CI)	(95% CI)		(95% CI)	
	*P*‐value	*P*‐value	*P*‐value		*P*‐value	
Nulliparous (*n* = 9321)						
Cephalic/cephalic	Ref	Ref	Ref		Ref	
Cephalic/non‐cephalic	1.07 (0.97–1.19) 0.425	1.04 (0.95–1.15) 0.159	1.03 (0.93–1.14) 0.079	^b^	1.06 (0.96–1.17) 0.137	^b^
Non‐cephalic/cephalic	5.90 (4.87–7.14) <0.001	5.91 (4.88–7.16) <0.001	5.98 (4.92–7.25) <0.001	<0	5.98 (4.93–7.25) <0.001	<0
Non‐cephalic/non‐cephalic	9.72 (8.00–11.79) <0.001	9.08 (7.48–11.04) <0.001	9.23 (7.59–11.23) <0.001	<0	10.01 (8.23–12.16) <0.001	<0
Parous (*n* = 12 490)						
Cephalic/cephalic	Ref	Ref	Ref		Ref	
Cephalic/non‐cephalic	1.16 (1.06–1.28) 0.002	1.14 (1.03–1.26) 0.010	1.13 (1.03–1.26) 0.016	7.14	1.18 (1.06–1.31) 0.002	<0
Non‐cephalic/cephalic	3.96 (3.43–4.59) <0.001	4.02 (3.45–4.67) <0.001	4.04 (3.47–4.70) <0.001	<0	4.40 (3.77–5.15) <0.001	<0
Non‐cephalic/non‐cephalic	8.99 (7.82–10.33) <0.001	8.53 (7.39–9.85) <0.001	8.62 (7.47–9.96) <0.001	<0	9.51 (8.20–11.03) <0.001	<0

*Note*: Model 1: adjusted for gestational age; model 2: adjusted for gestational age, maternal age, and maternal country of birth; model 3: adjusted for gestational age, diabetes, and pre‐eclampsia. For parous women, previous cesarean included in model 3.

Abbreviations: CI, confidence interval; Ref., reference group.

^a^ The percentage differences between model 1 and 2, and model 1 and 3 were calculated by the formula (OR model 1 − OR model *x*)/(OR model 1 − 1).

^b^ As the aORs were not significantly different between models 1, 2, and 3 for cephalic/non‐cephalic presentation for nulliparous, no calculation on difference was appropriate.

The findings for models 2 and 3 in Table 4 indicate that GA, maternal age, maternal country of birth, diabetes, PE, and previous CS had no or a minor effect on the association between twin presentation and intrapartum CS birth. It appeared that in parous women, maternal age, and country of birth explained 7.14% of the association between fetal presentation of cephalic/non‐cephalic and intrapartum CS birth (planned vaginal birth) (models 2 and 3 in Table 4).

### 3.3 Adverse maternal and fetal outcomes

The proportions of the planned delivery mode and actual delivery mode remained stable across the 30‐year study period, with only moderate changes (Figure S1).

Postpartum hemorrhage was more frequent among women with planned CS than among women with planned vaginal delivery (Figure S2), with aOR of 1.23 (95% CI 1.14–1.32).

The number of intrapartum stillbirths was 86, of which 67 (78%) occurred in preterm births <28 gestational weeks (Table S1). Frequency of stillborn newborns after 27 + 6 gestational weeks was extremely rare (in total, 19 cases) and therefore it was not appropriate to conduct meaningful analyses. Table S2 shows the frequency of intrapartum stillbirths by the twin presentations. In 15 twin births (30 newborns), both twins were intrapartum stillbirths, most of them before 28 + 0 weeks of gestation.

## 4 DISCUSSION

The majority of the twin births (83.4%) were planned as vaginal deliveries, and 16.6% were planned as CS, and this distribution remained stable during the 31‐year study period (Figure S1). Two out of three (65.9%) of all planned vaginal twin births were successful. Vaginal delivery was more often successful for parous women (72.9%) than for nulliparous women (56.5%). Non‐cephalic presentation of the first or both twins was less likely to result in successful vaginal delivery, and such cases were more often planned as CS compared with cephalic presentation of the first or both twins.

The present analysis of 31‐year real‐world data from the MBRN revealed that more than four out of five twin births were planned as vaginal deliveries, of which two out of three were successful vaginal deliveries. These results are consistent with a previous Dutch study of the planned and actual delivery modes in twin pregnancies, which found that 81% were planned as vaginal deliveries, of which four out of five were successful vaginal deliveries.^9^ Success rates for planned vaginal deliveries in single‐center population‐based studies from Finland and the United States were 81.6% and 74%, respectively, with planned CS rates of 29.2% and 51%.^2^, ^20^ In a multi‐center, population‐based study from France including twin pregnancies from GA 32 weeks onwards with a cephalic first twin, 75.4% were planned vaginal deliveries and these were successful in 80.3% of cases for both twins and associated with low composite neonatal mortality and morbidity.^21^

Only the study by Goossens et al. is comparable with our study with regards to sample size.^9^ In contrast to our study, which included all deliveries from GA 22 weeks, previous studies covered a narrower spectrum of GAs, such as only deliveries from GA 26, 32, or 35 weeks onwards.^2^, ^6^, ^9^, ^20^ Excluding deliveries that occur at lower GA is likely to reduce the generalizability of the results in clinical settings.^2^, ^6^, ^9^, ^20^

In our study, the data were stratified by parity, whereas many previous studies have not distinguished between fetal twin presentations with regard to parity.^6^, ^8^ Our results indicate that the planned delivery mode was not affected by parity, but parous women were more likely than nulliparous women to have a successful vaginal delivery (72.9% and 56.5%, respectively). Approximately one out of six women in our study had a planned CS regardless of parity, which is lower than the proportions of planned CS rates in previous studies.^2^, ^6^, ^8^, ^9^, ^20^

In our study, the prevalence of combined delivery (first twin vaginal and second twin CS) was 3.3%, whereas Goossens et al. reported 4.4%^9^ and Aviram et al. 5.4%.^22^ This suggests that the Norwegian delivery‐mode recommendations do not lead to a higher rate of combined twin deliveries.

Our results show that the planned delivery mode was strongly affected by the fetal presentation, with twins in cephalic/cephalic presentation being most likely to be born vaginally and the vaginal delivery rate being lowest for twins in non‐cephalic/non‐cephalic presentation (Table 1). The partially adjusted logistic regression models showed that maternal demographics (age, country of birth) had no or a minor effect, and pregnancy complications and previous CS had no effect on the association between twin presentations and planned CS or intrapartum CS (Tables 3 and 4, model 2). Approximately 10% of twin pregnancies with cephalic presentation of the first or both twins and 30%–40% of twin pregnancies with non‐cephalic presentation of the first or both twins were planned as CS (Table 2). The latter finding is in line with many previous studies in which a non‐cephalic first twin was an indication for a planned CS.^2^, ^9^, ^20^ More than half of the twins with non‐cephalic presentation of the first or both twins in the Dutch study were planned as CS, and vaginal delivery was successful in 30%–40% of those planned as vaginal delivery.^9^

In Norway, the overall CS rate has remained stable and lower than in many other countries worldwide.^4^, ^5^ Only moderate changes in CS rates (planned or intrapartum) were observed during the study period, with no discernible increasing trend. Women with planned CS had PPH more often compared with women with planned vaginal delivery. The intrapartum stillbirth rate was extremely low, at 0.16% (86 of 52 286 newborns). These findings indicate that the Norwegian management of twin births is a safe option.

A strength of this study was the population‐based real‐world data, encompassing all twin births regardless of GA, providing robust and generalizable results compared with studies with selected populations.^23^ Data from the mandatory MBRN is considered reliable, particularly regarding the number of fetuses and mode of birth.^24^

The MBRN did not record CS indications, preventing analysis of specific reasons such as dystocia, fetal hypoxia/asphyxia, chorionicity, or maternal request. However, Norwegian National Obstetric Guidelines recommend labor induction at 36–37 weeks of gestation for monochorionic diamniotic twins, without specifying the delivery mode, whereas CS at 32–34 weeks of gestation is recommended for monochorionic monoamniotic twins.^3^

## 5 CONCLUSION

More than half of women with a twin pregnancy had a successful vaginal delivery. The most important risk factor for both planned and intrapartum CS was the first twin in non‐cephalic presentation regardless of the presentation of the second twin.

Strong recommendations for planned CS restrict the choices that women have for the delivery mode. Keeping the option of the vaginal route as the primary mode of delivery in a twin pregnancy maintains the right of the woman to have a vaginal delivery and avoid a uterine scar.

## AUTHOR CONTRIBUTIONS

Meryam Sugulle and Katariina Laine conceived and planned the study. Katariina Laine performed the analyses. Meryam Sugulle, Katariina Laine, and Sari Räisänen primarily wrote the manuscript. All authors were involved in the interpretation of the results, and in drafting and reviewing the manuscript. All authors revised the manuscript and accepted the submitted version.

## FUNDING INFORMATION

The Norwegian SIDS and Stillbirth Society provided financial support (grant number 554.04/14) to retrieve data files from the registry.

## CONFLICT OF INTEREST STATEMENT

The authors have no conflicts of interest.

## Supporting information


Figure S1.

Figure S2.

Table S1.

Table S2.

Data S1.

## Data Availability

Research data are not shared.
