# Supplementary figures and images for: Effects of fetal presentation on mode of delivery in 26 143 twin pregnancies: A nationwide, population‐based observational study of 31‐year real‐world data
Source: Int J Gynaecol Obstet. 2025 Mar 29;170(3):1309–16. doi: 10.1002/ijgo.70103 (PMC12374014; doi:10.1002/ijgo.70103)

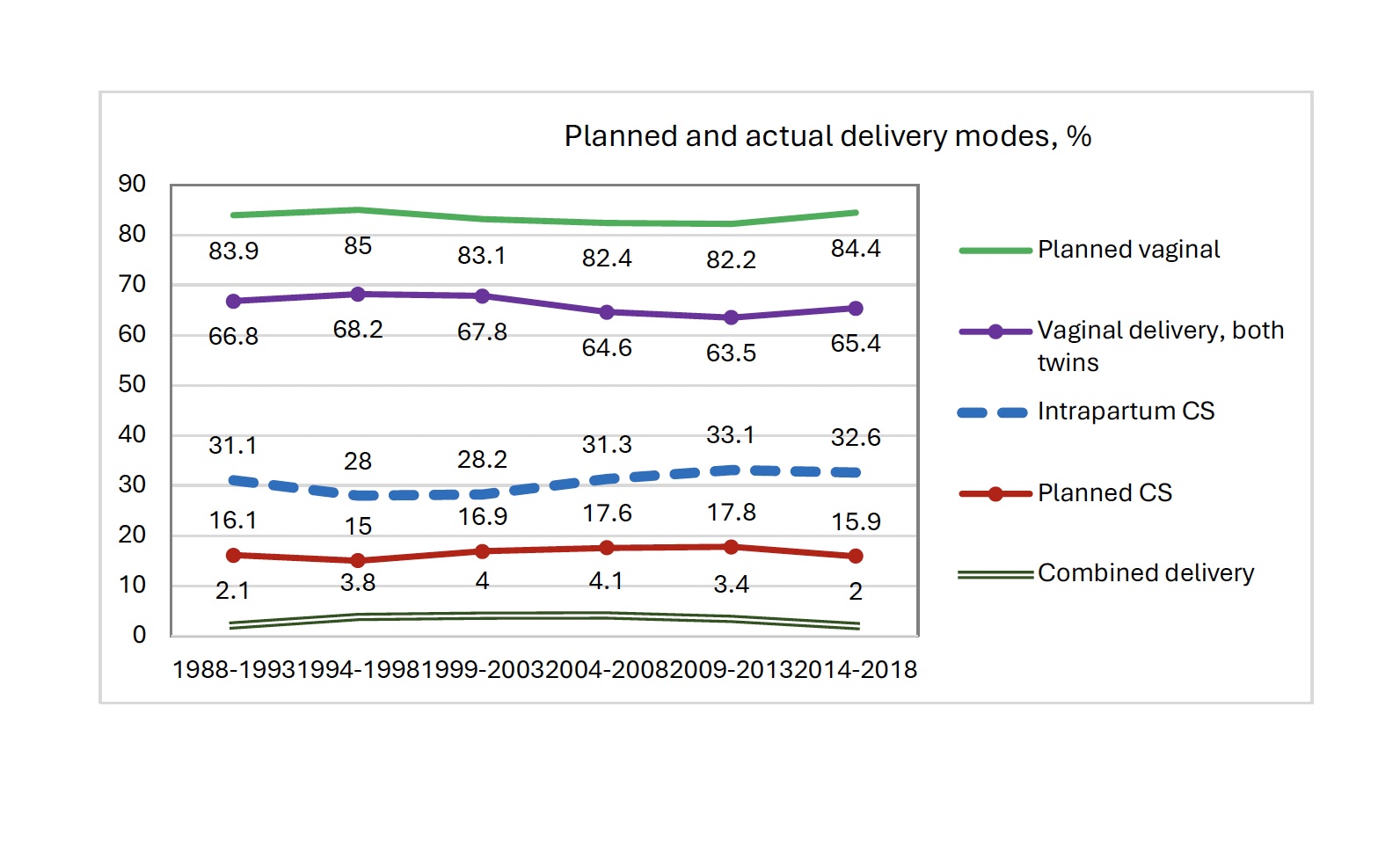

Supplement: Supplementary file 1 — Figure S1. [file IJGO-170-1309-s001.jpg]

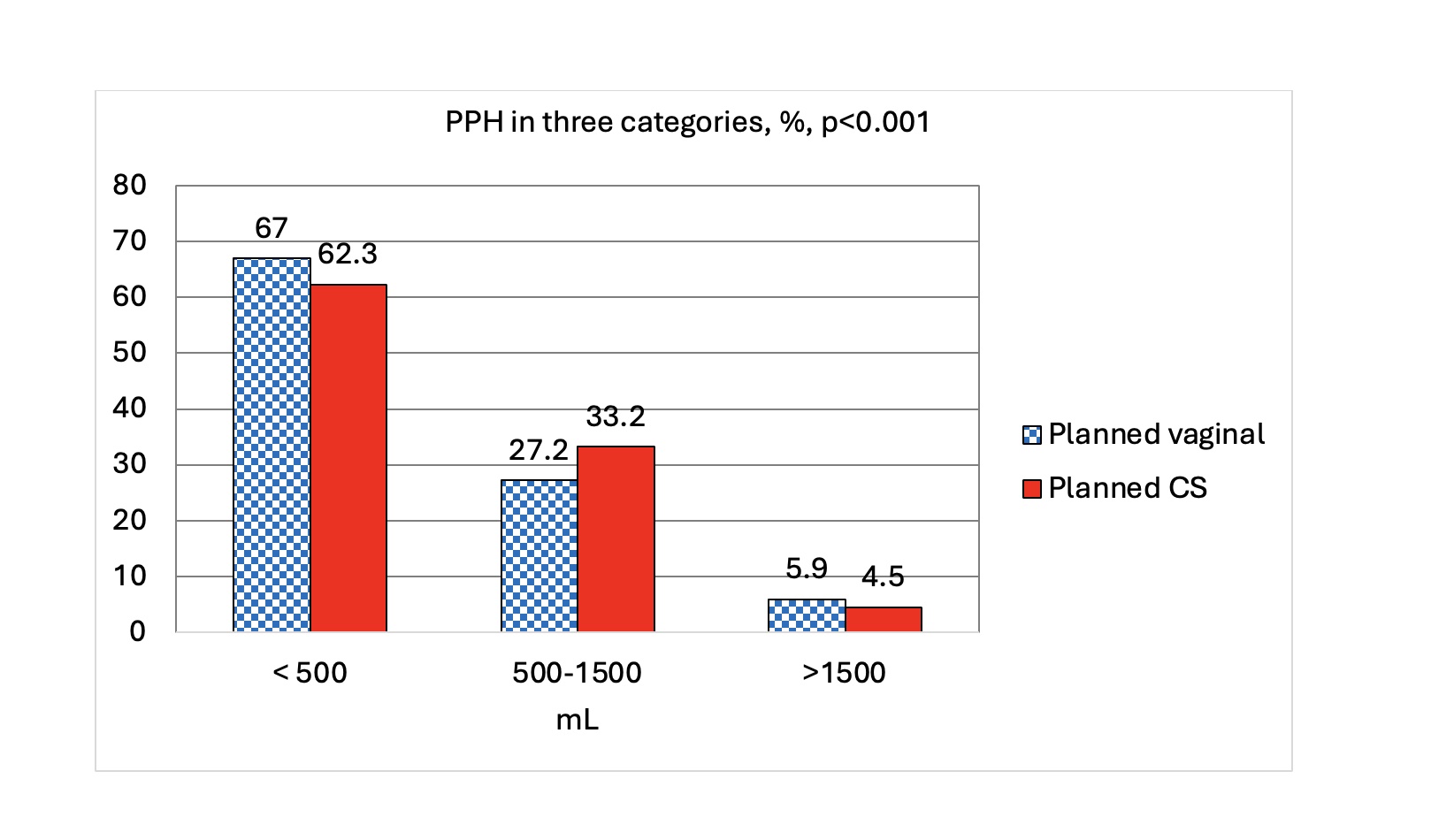

Supplement: Supplementary file 2 — Figure S2. [file IJGO-170-1309-s002.jpg]
