# Supplementary material for: Effects of fetal presentation on mode of delivery in 26 143 twin pregnancies: A nationwide, population‐based observational study of 31‐year real‐world data
Source: Int J Gynaecol Obstet. 2025 Mar 29;170(3):1309–16. doi: 10.1002/ijgo.70103 (PMC12374014; doi:10.1002/ijgo.70103)
Supplement: Supplementary file 3 — Table S1. [file IJGO-170-1309-s003.docx]

**Table S1.** Number of babies with intrapartum stillbirth according to gestational age (n=26 143 pregnancies, 52 286 babies). The exact number of cases is not reported in groups less than 5, to protect the individuals’ privacy rights according to the European Union’s General Data Protection Regulation.

| **GA** | Planned vaginal delivery | Planned CS | Total  n=86 of 52 286 |
| --- | --- | --- | --- |
| **22+0 to 27+6 weeks** |  |  |  |
| First twin | 34 | 0 | 34 |
| Second twin | 33 | 0 | 33 |
| **28+0 to 33+6 weeks** |  |  |  |
| First twin | 5 | 0 | 5 |
| Second twin | less than 5 | 0 | less than 5 |
| **34+0 to 36+6** |  |  |  |
| First twin | less than 5 | 0 | less than 5 |
| Second twin | less than 5 | less than 5 | less than 5 |
| **37+0 or more** |  |  |  |
| First twin | less than 5 | less than 5 | less than 5 |
| Second twin | less than 5 | 0 | less than 5 |

CS: Cesarean section; GA: Gestational age
