# Supplementary material for: Effects of fetal presentation on mode of delivery in 26 143 twin pregnancies: A nationwide, population‐based observational study of 31‐year real‐world data
Source: Int J Gynaecol Obstet. 2025 Mar 29;170(3):1309–16. doi: 10.1002/ijgo.70103 (PMC12374014; doi:10.1002/ijgo.70103)
Supplement: Supplementary file 4 — Table S2. [file IJGO-170-1309-s005.docx]

**Table S2.** Number of babies with intrapartum stillbirth according to fetal presentation (n=26 143 pregnancies, 52 286 babies). The exact number of cases is not reported in groups less than 5, to protect the individuals’ privacy rights according to the European Union’s General Data Protection Regulation.

|  | Intrapartum stillbirth | | |
| --- | --- | --- | --- |
|  | First twin | Second twin | Both twins |
| **Presentation** |  |  |  |
| Both Cephalic | 14 | 13 | 14 |
| Cephalic/non-cephalic | less than 5 | 10 | less than 5 |
| Non-cephalic/cephalic | 6 | less than 5 | less than 5 |
| Non-cephalic/non-cephalic | less than 5 | less than 5 | 8 |
