# Supplementary material for: Effects of fetal presentation on mode of delivery in 26 143 twin pregnancies: A nationwide, population‐based observational study of 31‐year real‐world data
Source: Int J Gynaecol Obstet. 2025 Mar 29;170(3):1309–16. doi: 10.1002/ijgo.70103 (PMC12374014; doi:10.1002/ijgo.70103)
Supplement: Supplementary file 5 — Data S1. [file IJGO-170-1309-s004.docx]

**Figure S1.** Prevalence (%) of planned and actual delivery mode by six time periods shown as years.

CS=cesarean section

**Figure S2**. Prevalence (%) of postpartum haemorrhage in milliliters in three categories by planned delivery mode.

CS=cesarean section; mL= millilitres; PPH=postpartum hemorrhage
